# The Compensatory Protective Effects of Social Support at Work in Presenteeism During the Coronavirus Disease Pandemic

**DOI:** 10.3389/fpsyg.2021.643437

**Published:** 2021-03-23

**Authors:** Jia Wun Chen, Luo Lu, Cary L. Cooper

**Affiliations:** ^1^Department of International Trade, Chihlee University of Technology, New Taipei City, Taiwan; ^2^Department of Business Administration, National Taiwan University, Taipei, Taiwan; ^3^Alliance Manchester Business School, University of Manchester, Manchester, United Kingdom

**Keywords:** sickness presenteeism, supervisory support, collegial support, innovative behavior, well-being, conservation of resource, cultural values

## Abstract

The present study investigated the lasting effects of sickness presenteeism on well-being and innovative job performance in the demanding Chinese work context compounded with the precarities of the post-pandemic business environment. Adopting the conservation of resources (COR) theory perspective, especially its proposition of compensation of resources, we incorporated social resources at work (supervisory support and collegial support) as joint moderators in the presenteeism–outcomes relationship. We employed a panel design in which all variables were measured twice with 6 months in between. Data were obtained from 323 Chinese employees working in diverse industries in Taiwan. We found that after controlling for the baseline level of well-being, presenteeism did not have a lasting effect on employees' exhaustion. However, presenteeism did have a negative lasting effect on employees' innovative behavior 6 months later. Moreover, we found a significant three-way interaction of presenteeism, supervisory support, and collegial support on employees' innovative job performance, after controlling for the baseline level of performance. Specifically, when working under illness, employees displayed the best innovative performance with high levels of both supervisory and collegial support, the worst performance with both support being low, and the intermediate when any one of the support being high. This can be taken as the preliminary evidence to support the COR proposition of resource caravans, showing that supervisory support and collegial support compensated for each other as critical resources in alleviating the impact of working under sickness on employees' innovative performance. Theoretical implications of the findings are discussed, taking into account the macro-cultural context of the East Asian Confucian societies. We also reflected on the managerial implications of the lasting damages of sickness presenteeism and benefits of mobilizing social resources on employees' well-being and performance.

## Introduction

Year 2020 has witnessed the unprecedented triple pandemic rampaging the entire world, e.g., the health crisis of coronavirus disease 2019 (COVID-19), the economic recession caused by restrictions and lockdowns, and the social revolution triggered by amplified social injustices when the going gets tough. In the post-pandemic era, the consolidating mainstream values of well-being, equity, diversity, and inclusion call for concerted efforts from academics in the creation, communication, and application of scientific knowledge. In the post-pandemic business world, repeated lockdowns and the continuing “working from home” practice have blurred the demarcation between the work and home space, causing more excessive engagement in work activities (Cigna, 2020). While western countries are still fighting to control the upsurge of pandemic, Taiwan acted swiftly at the very beginning of the pandemic (early 2020) by sealing its borders, banning large gatherings, and mandating wearing of face masks. Consequently, Taiwan has succeeded in holding the death toll in single digit (Taiwan Centers for Disease Control, 2020) and largely maintained a “normal life” with no substantial restrictions on economic and social activities. Nonetheless, heavily reliant on export and deeply embedded in the global value chains, business outlook in Taiwan is uncertain; thus, fear for prolonged economic recession and resultant job insecurity are heightened among employees (Lee et al., 2017). Facing the precarities of the post-pandemic business environment, employees are compelled to commit more excessive work behaviors to protect job prospects and to catch up with increasing work demands. One common form of the excessive availability for work is sickness presenteeism (SP) (Cooper and Lu, 2019). SP (or presenteeism, hereafter used interchangeably) is the phenomenon of people who despite ill health that should prompt rest and absence from work are still turning up to their jobs (Aronsson et al., 2000). In the organizational research field, researchers now agreed that SP denotes to the *behavior* of going to work when sick (Johns, 2012). Responding to the post-pandemic challenges and the job insecurity pressures, we expect that the presenteeism behavior will become more prevalent in the West and the East, and its noxious effects will compound the generic post-pandemic challenges on individual well-being and organizational effectiveness. Thus, the present study aimed to clarify the *lasting* negative consequences of the behavior on the individual's well-being and job performance, which in aggregate contributes to organizational effectiveness. Further, contributing to the inclusiveness of scientific contents, our study targeted the under-represented Asian populations in the extant presenteeism literature. This is because in the East Asian societies, presenteeism is more prevalent and poses graver impacts on the employees. Lu et al. (2013a) found that Taiwanese employees reported significantly higher rates of presenteeism, and consequently suffered greater exhaustion and lower job satisfaction, compared with their British counterparts.

Whether employees force themselves to attend work out of fear for losing their jobs, succumbing to mounting work demands following the pandemic and recession, or honoring the cultural morals of “hard work,” presenteeism can lead to exhaustion (Demerouti et al., 2009) and may be even costlier than absenteeism to employers due to reduced productivity (Hemp, 2004; Burton et al., 2006). Mobilizing valuable resources to alleviate the negative effects of working under illness is of paramount importance in the current changing work environment. In the conservation of resources (COR) theory, resources are broadly defined as those entities that either are centrally valued in their own right or act as a means to obtain centrally valued ends (Hobfoll, 2002, p. 307). Resources thus include a wide range of tangible and intangible things (e.g., physical, material, cognitive, motivational, social, and emotional) that all are inherently valuable. Social support is one of the most valuable resources in coping with work stress and can replace or reinforce other absent resources (Hobfoll, 1989). Stemming from the motivational facet of COR theory, individuals strive to obtain and retain multiple resources from different social networks to prepare themselves for potential future losses. We thus set out to explore how different sources of social support at work act as a resource caravan to alter the impact of SP on employees' strain and job performance. No study to our knowledge has investigated simultaneously the protective effects of collegial support and supervisory support in the SP situation, to empirically test the COR proposition of resource caravan, specifically resource compensation. This is an important oversight because employees have vertical (i.e., supervisory support) and horizontal relationships (i.e., collegial support) at work and thus have the option of mobilizing and investing different resources to cope with situational demands.

**Figure 1:**
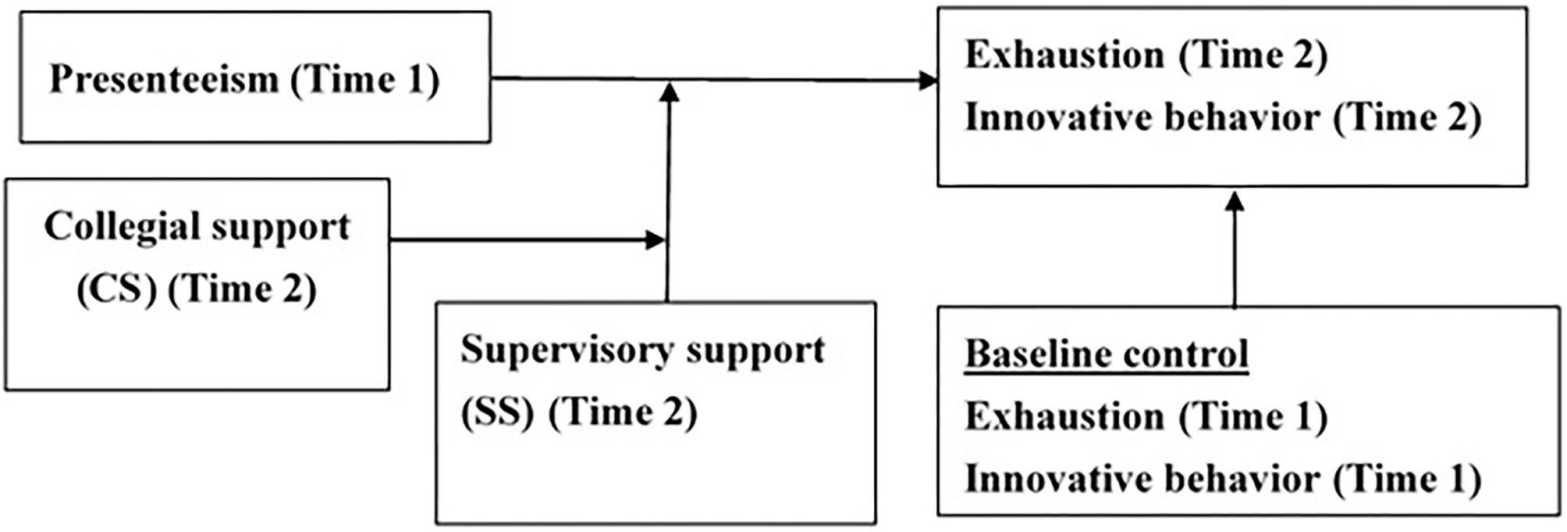
The research model.

To sum, the thrust of this study is 2-fold. First, we aimed to clarify the lasting effects of working under illness on employees' well-being and innovative performance. Such claims have often been made in the extant literature without rigorous scientific evidence to endorse, largely due to the scarcity of longitudinal studies (Johns, 2012; Karanika-Murray and Cooper, 2018; Lohaus and Habermann, 2019). In particular, employees' innovative performance has rarely been investigated as a work outcome in the presenteeism research (Fan, 2018). Second, we aimed to examine the joint effects of supervisory support and collegial support in the presenteeism–work outcomes relationship. Applying the resource caravan proposition of COR theory, we focused on the compensatory effects of the dual social support on alleviating the impact of presenteeism on employees' well-being and innovative performance. By investigating the medium-term effects of sickness presenteeism on both exhaustion and innovative performance and, at the same time, by analyzing the compound effects of two kinds of social support, we believe that this study moves a step forward in the existing literature on presenteeism. The topic under consideration is of the utmost importance for organizations too. Furthermore, conducting our research in a Chinese society (Taiwan), the present study will enrich our cultural understanding of the presenteeism behavior in the cultural context of hard working and perseverance. Figure 1 is the graphical representation of our research model.

## Hypothesis Development

COR theory proposed that stress occurs (a) when the central or key resources are threaten with loss; (b) when the central or treasured resources are lost; or (c) when there is a failure to gain centrally or key resources following significant efforts. In case of resources lost, an individual strives to obtain, retain, foster, and protect core value and resources to fend against work demands or life stress (Hobfoll et al., 2018). Hobfoll (1989) proposed the concept of resource caravans arguing that resources “run in packs” (Hobfoll, 2012), even though they may have distinct theoretical origins. However, the nature of resource caravans in COR theory is still not well-developed (Hobfoll, 2002, 2011), and we know little about *how* resources combine or compensate one another to meet personal goals, or *when* such resource combinations or compensations take effect. Acquiring new resources will give individuals a sense that they are capable of meeting stressful challenges, and in turn, they will become more confident in deploying resources and investing in gaining more resources (Hobfoll et al., 2018). Building on the idea of resource caravans, we focused on supervisory support as a salient work feature for the Chinese employees to examine a specific form of resource caravan; namely, when a key social resource is low or absent (lack of support from the supervisor), a second resource (support from co-workers) may substitute for it and perform the compensatory role in coping.

### The Lasting Damages of Presenteeism: Working Under Illness as a Depletion in Resource

Cooper (1996) originally defined presenteeism as being physically present but functionally absent, implying a reduction in individual productivity while working under suboptimal health conditions. Subsequent organizational researchers too mostly approach the presenteeism behavior as a decision option (against absenteeism) when employees are faced with “to go or not to go” choices precipitated by an ill-health event (Johns, 2010; Halbesleben et al., 2014; Miraglia and Johns, 2016; Gosselin, 2018). Not surprisingly, the bulk of the presenteeism research has focused on antecedents and correlates of the behavior, while empirical research on the outcomes of presenteeism is still sparse (see reviews by Miraglia and Johns, 2016; Karanika-Murray and Cooper, 2018; Lohaus and Habermann, 2019). Another lacuna of the organizational research on presenteeism is the scarcity of longitudinal studies demonstrating lasting effects (positive or negative) on employees' well-being and job performance (see reviews by Miraglia and Johns, 2016; Cooper and Lu, 2019; Lohaus and Habermann, 2019). While cross-sectional studies have found that presenteeism is negatively associated with the concurrent employees' health, work attitude, job performance, and innovation (Lu et al., 2013b; Conner and Silvia, 2015; Miraglia and Johns, 2016), we have little insight into the lasting effects of presenteeism on individuals (Demerouti et al., 2009; Lu et al., 2013b, 2014; Skagen and Collins, 2016) and the processes that change the outcomes of the behavior. More research on the dynamic relationship between presenteeism and employees' work outcomes, especially the trajectory over time, is thus needed to distinguish the assumed negative outcomes of the behavior (*bad presenteeism*) (Cooper, 1996; Hemp, 2004; Demerouti et al., 2009; Lu et al., 2013b) from the purported positive outcomes (*good presenteeism*) (Ashby and Mahdon, 2010; Miraglia and Johns, 2016).

Viewed from the COR perspective, sickness presenteeism represents a scenario for resource depletion (Ferreira, 2018). COR theory relies centrally on the differential effects of objective and cultural contexts on determining the stress process (Hobfoll, 2001). Specifically, individuals strive to obtain, retain, foster, and protect their resources not only in case of resource lost but also in normal time to prepare themselves to deal with potential future losses (Hobfoll et al., 2018). For employees, stress can come from working under illness. By precluding the possibility of recovery, excessive work behaviors including long hours and working through illness induce sustained negative activation, soliciting the constant “feelings of tension and distress” (Hahn et al., 2012), which causes detrimental effects on the psychobiological system (Ursin and Eriksen, 2004). Research has found that working long hours (Lu and Chou, 2020) and inability to detach after work (Sonnentag and Fritz, 2015) trigger continuous resource loss leading to lasting strain. Few longitudinal studies have also found that working while ill predicted future poor self-rated general health, but the findings were less clear when specific measures of physical health were used (Skagen and Collins, 2016). Studies with Chinese workers produced mixed results, as presenteeism was associated with well-being measures in a 2-month (Lu et al., 2013b) but not 3-month (Lu et al., 2014) follow-up. It seems that presenteeism may constitute a hazard for the individual's quality of life, but not necessarily precipitate specific health problems. Also, clear research on the incubating time frame is need. We thus focused on a subjective indicator of well-being, exhaustion, as the likely outcome of the sustained negative activation of working under sickness. Demerouti et al. (2009) showed a positive reciprocal relationship between presenteeism and exhaustion for Dutch nurses, indicating that working while sick increases exhaustion that, in turn, raises the likelihood of presenteeism. Lu et al. (2013b) also found evidence of a reciprocal relationship between presenteeism and exhaustion in a heterogeneous sample of Chinese employees, using a different measure of presenteeism. As a generic stress theory, exhaustion as an indicator of strains is one of the most studied outcome variables in the COR literature (Hobfoll, 2011). We thus hypothesized:

*H1a: Presenteeism at T1 will be positively related to employees' exhaustion at T2*.

Although presenteeism is viewed as a precursor to decreased performance, thus productivity loss for organizations (Hemp, 2004; Burton et al., 2006; Halbesleben et al., 2014), there is surprisingly little empirical research on the relationship between the two. The available but also inconclusive research has highlighted a weak or non-existent relationship between presenteeism and job performance (Munir et al., 2005; Johns, 2011; Lu et al., 2013b, 2014). Even rarely examined is the employees' performance on innovation, separate from in-role task performance. Employees' innovative behavior involves both the generation of new ideas and the subsequent stages of internal promotion and implementation of such ideas (Anderson et al., 2014; De Clercq et al., 2016). Innovative behavior of employees is an important aspect of work performance, which is intricately linked to organizational innovation and competitiveness (Amabile et al., 1996; Yuan and Woodman, 2010). COR theory purports that individuals strive to obtain, retain, foster, and protect their resources in stress and coping (Hobfoll et al., 2018). However, working under illness hampers the recovery of vital physical and psychological resources. Presenteeism is found to be associated with certain psycho-affective states, such as low energy, negative affect (Gustafsson and Marklund, 2011), depression, and anxiety (Lu et al., 2013b; Conway et al., 2014), which are harmful to employees' creativity. Empirical research has further demonstrated that presenteeism hampered cognitive functioning and negatively affected brainstorming, concentration, and both the quantity and quality of work produced (Hansen and Andersen, 2008). In a rare empirical examination of the direct relationship between presenteeism and employees' innovative behavior, Fan and Lu (2020) found a U-shaped trajectory moderated by the psychological drives to commit the presenteeism behavior. Specifically, they noted that the positive drives (e.g., for professionalism and career promotion) enhanced the U-shape relationship (making it steeper), while the negative drives (e.g., for fear of loss and social criticism) weakened the U-shape relationship (making it flatter). Viewed from the COR perspective, working while unwell requires more effort to maintain the expected level of performance, as employees need to increase concentration and cognitive labor to overcome the distracting symptoms of illness. In such a resource hemorrhage circumstance, employees may have to conserve valuable energy and brain power to maintain performance on in-role tasks, leaving little resources for the “above and beyond” innovative performance. Thus, we hypothesized:

*H1b: Presenteeism at T1 will be negatively related to employees' innovative behavior at T2*.

### The Resource Compensation Mechanism: Interactive Effects of Social Support at Work

According to COR theory, an individual would try to gain other resources to protect against resources loss and strain (Hobfoll et al., 2018). *Guanxi*, Chinese for “relationships,” which equals to the concept of human capital or social support, is an invisible but critical resource in the collectivistic cultural context and could buffer or exacerbate the relationship between presenteeism and work outcomes (Lu et al., 2013a; Glazer and Amren, 2018). Workplace social support is not a monolithic construct but rather emanates from multiple sources, including supervisors, coworkers, and employing organization (Halbesleben, 2006; Kossek et al., 2011), and may have different effects on individual behavior and outcomes. Although past studies have linked supervisory support to positive work behavior (Gilbreath and Benson, 2004; Rad and Yarmohammadian, 2006) and documented the beneficial effect of collegial support in employee retention in the organizational literature, little research has explored how different kinds of social relationships might interactively affect employees' job performance and strains. In other words, we know little about *how* resources combine to meet personal goals, or *when* resource combinations take effect (Hobfoll, 2002, 2011). This is what the concept of “resource caravans” in the COR aims to explain (Hobfoll, 1989, 2012). Furthermore, while past studies focused exclusively on personal motivational factors, innovative behavior is the joint outcomes between the individual and the situation (Amabile et al., 1996). We thus include social resources at work as situational moderators in the presenteeism–innovation relationship.

Resources “run in packs” and interact with one another is arguably the least developed and rarely tested theoretical proposition in the COR framework. Building on the idea of resource caravans, we focused on social support at work for employees working under illness to examine a specific form of resource caravan; namely, when a key social resource is low or absent (e.g., lack of support from the supervisor), a second resource (e.g., support from colleagues) may substitute for it and perform the compensatory role in coping. Brunner et al. (2019) found that job and personal resources can buffer the negative effects of job stressors (time pressure, performance constraints, work overload, or task uncertainty) on health-related productivity losses caused by presenteeism and absenteeism. Furthermore, they also found the compensatory effect of job resources for employees with low personal resources facing high job stressors.

Applying the resource caravan perspective, employees have to work and communicate with supervisors and colleagues in the workplace; thus, the support from supervisors and coworkers could be concurrently mobilized and jointly affected. However, the *interactive* effects of these two most salient forms of social support on the stressor–outcomes relationships are rarely discussed and empirically examined. The extensive literature in stress and coping has shown that social support gained from different sources can have different implications for coping. For example, when coping with demands of the work and family dual role, supervisory support was more useful in reducing the work and family conflict (negative spillover), while spousal support was more instrumental in creating the work and family enrichment (positive spillover) (Lu and Chang, 2014).

In the work context, supervisory support as an indicator of good leader–member relationship is crucial for career advancement and good quality of work life, such as satisfaction and engagement (Karimi and Nouri, 2009; Karimi et al., 2011). However, coworker friendship as an indicator of social embeddedness and comradeship is also vital for workplace social integration and well-being (Chiaburu and Harrison, 2008; Poon, 2011; Zaitouni and Ouakouak, 2018). Research has found that when working under illness, supervisory support buffered the negative impact of presenteeism on employees' exhaustion (Lu et al., 2013a). This is because supervisors can decide how to allocate resources in the workplace. Thus, when presenteeism was triggered by organizational constraints such as heavy workload or shortage of manpower, the instrumental value of supervisory support would be realized if workload could be adjusted or supplementary manpower assigned. However, in the present environment of post-pandemic recession and cut down, removing the organizational constraints or granting work flexibility is often not the managers' discretion. At such testing times, the value of support from other sources for instance, those close at work, would be amplified for coping with the noxious effects of demanding work. This dynamism of resource mobilization from different sources at work is unraveled in a qualitative study with nurses interviewed in focus groups (Dew et al., 2005). Some nurses used a metaphor of “sanctuary.” When they had to work while ill, they were caringly helped by their “family.” Consequently, they were able to work through mild sickness and eventually felt better or ignored discomfort altogether. It is likely that when the individual is caught in a continuous resource depletion situation (e.g., working through illness), in addition to (or lacking) supervisory support, mobilizing support from the colleagues and gain help or comfort from whom close at work may compensate for the loss or absence of other resources.

This is in line with the notion of “resource caravan” in the COR: resources exist in groups and clusters within the ecological realm, and those with greater resources are less vulnerable to resource loss and more capable of resource gain (Hobfoll et al., 2018). It thus seems that when social resources are mobilized from all corners and sources at work, employees may be better equipped to cope with the noxious effects of presenteeism, thus containing its impacts on individual well-being and performance. More importantly, resources can foster the gain or loss spirals, and this is why individuals with low levels of resources are less able to achieve resource gains (e.g., they do not have enough resources to invest). To prevent and prepare for future resources lost, an individual tends to create more potential resources (e.g., strengthen bond and sense of comradeship with colleagues) and help employees to cope and adapt in the context of presenteeism (Lu et al., 2013a). Acquiring new resources will give individuals a sense that they are capable of meeting stressful challenges, and in turn, they will become more confident in deploying resources and investing in gaining more resources (Hobfoll et al., 2018). Conversely, individuals who lack resources are more vulnerable to resource loss and less capable of resource gain. Research has indeed shown that employees with more resources are more adaptive and can solve job- and career-related difficulties and achieve their personal goals more successfully than those with fewer resources (Hobfoll, 2002). We thus expect that individuals with the abundance of resources (e.g., high on both supervisory and collegial support) will cope the best while working through sickness, showing the *least* damaging effects on well-being and performance. Those having the minimal resources (e.g., low on both supervisory and collegial support) will suffer the most severe blow on well-being and performance when working under sickness. To demonstrate the resource caravans idea, we expect that individuals with high levels of either supervisory support or collegial support can use it as a second resource when primary resource depletion is high (lack of collegial support or supervisory support) and, thus, are compensated to a certain extent for the negative consequences of working through illness. More precisely, the relationship between presenteeism and individual outcomes (i.e., exhaustion and innovative performance) will vary depending on the individual's level of supervisory and collegial support, thereby demonstrating a pattern of moderated moderation. We thus hypothesized:

*H2a: A three-way interaction of presenteeism and social support from supervisor and colleagues is related to employees' exhaustion. Specifically, in sickness presenteeism, the employee exhaustion is at the lowest level when supervisory support and collegial support are both at high levels; at the highest level when supervisory support and collegial support are both at low levels; and at the intermediate level when one of the supports is high and the other is low*.

*H2b: A three-way interaction of presenteeism and social support from supervisor and colleagues is related to employees' innovative behavior. Specifically, in sickness presenteeism, the employee innovative behavior is at the highest level when supervisory support and collegial support are both at high levels; at the lowest level when supervisory support and collegial support are both at low levels; and at the intermediate level when one of the supports is high and the other is low*.

## Method

### Procedure

As the majority of the existing studies on presenteeism employed a cross-sectional design, we are unable to generate comprehensive knowledge on the prospective effects of presenteeism on performance and well-being (Lohaus and Habermann, 2019). We thus employed a two-wave panel design in which all variables were measured twice with the interval of 6 months. While there is a constant call for more longitudinal study designs, there is no consensus for the optimal time lag (Dormann and Griffin, 2015). As the Demerouti et al. (2009) looked at the long-term effect (time frame of 1.5 years) and Lu et al. (2013b) looked at the short-term effect (time frame of 2 months), we in the present study adopt a medium-term time frame of 6 months, allowing sufficient time for presenteeism to incubate its effects on job performance and well-being. Our sample was composed of full-time employees working in different organizations and diverse industries in Taiwan. The only inclusion criterion was “working” during the study period (July 2019 to April 2020). As normal life in Taiwan was largely undisrupted in the COVID pandemic, none of our participants were on furlough scheme or working from home. We did not include foreign nationals or migrant workers; thus, our sample was all ethnic Chinese. The study was approved by Research Ethics Committee of the principle researcher's institute. Individual written informed consent for participation was not required for this study in accordance with the national legislation and the institutional requirements. The paper-pencil survey was carried out using convenient sampling to recruit participants through personal contacts of the researchers. Some participants were enrolled in executive education programs, and others were recruited through managers in various organizations. At Time 1 (T1), a cover letter accompanied the questionnaire, explaining the aims of our study and assuring confidentiality. Participants filled in questionnaires in their leisure and returned them in sealed envelopes to their contact persons or directly to the researchers. The initial survey was completed by 682 persons (response rate: 96.46%), with 631 persons providing usable data. Six months later, following the same procedure, 407 persons completed the survey again (T2, retention rate of 64.81%). By matching the code self-generated by respondents at T1, the T1 and T2 data from 333 persons were combined. The “match code” was only known to the participant, not disclosed to the researcher; thus, the questionnaire remained anonymous. We further excluded those with excessive missing data (more than 1/3) on the core variables, resulting in the final sample size of 323. We examined the attrition bias by comparing the participants in the panel sample and the dropouts on demographic characteristics and mean scores of all variables (T1). We found no significant differences in any variables, indicating no serious attrition bias.

All of our participants were white-collar workers. The sample was 33.7% male and 66.3% female, with a mean age of 36.91 (SD = 8.89, range = 20–65) and mean job tenure of 7.25 years (SD = 6.57). Just over half of the sample (54.2%) were married. Most of the sample had education above college level (96%), and more than a quarter of the respondents (28.8%) were managers. We asked participants to report the size of their organizations in three categories, employing under 250 people, between 251 and 1,000, and over 1,000. Data showed that our participants equally distributed in small- and medium-sized enterprises (SMEs) (35%, under 250 employees) and large companies (37.50%, over 1,000 employees). We also asked participants to identify the industries of their organizations and found service (25.50%), manufacturing (21.10%), education/culture (14.80%), and finance (10.80%) as being the top four industries. In Taiwan, these sectors are slightly affected by the COVID pandemic and maintained normal operations throughout.

### Measures

The structured questionnaire was written in Chinese, and all the standard measures have been used and validated with Chinese samples in previous studies (the Chinese validation reference is given for each scale below).

#### Presenteeism

We used the two-item presenteeism scale developed and validated for the Chinese populations by Lu et al., 2013a, 2014) to measure the act of “sickness presenteeism” (e.g., “Although you feel sick, you still force yourself to go to work”). With a time frame of “past 6 months,” four-point scales were used (0 = Never, 6 = More than five times) to rate the frequency of presenteeism behavior. The internal consistency reliability of the scale was 0.85 (T1) and 0.86 (T2) in the present study.

#### Supervisory Support

We used the four-item Supervisor Support Scale developed by O'Driscoll et al. (2004; for the Chinese version: Lu and Chang, 2014). Respondents were asked how often they had received four different types of support from their supervisors: helpful information or advice, sympathetic understanding and concern, clear and helpful feedback, and practical assistance. Six-point frequency scales were used (1 = Never, 6 = Very frequently). The internal consistency reliability of this scale was 0.96 (T1) and 0.96 (T2) in the present study.

#### Collegial Support

We used the six-item Workplace Friendship Prevalence Scale developed by Nielsen et al. (2000; for the Chinese version: Mao, 2006) to measure the prevalence of workplace friendship (e.g., “I have formed strong friendships at work”). Five-point agreement Likert scales were used (1 = Completely disagree to 5 = Completely agree). The internal consistency reliability of this scale was 0.83 (T1) and 0.85 (T2) in the present study.

#### Exhaustion

According to past research, emotional exhaustion is the core component of burnout compared with other dimensions (depersonalization and personal accomplishment) and the most obvious manifestation of the syndrome (Taris et al., 2005). We used a nine-item emotional exhaustion scale from the Maslach Burnout Inventory (Maslach and Jackson, 1986; for the Chinese version: Lu et al., 2013b) to measure exhaustion (e.g., “I feel used up at the end of the workday”). Seven-point scales were used (0 = Never experienced such a feeling to 6 = Experienced such feelings every day). The internal consistency reliability of the scale was 0.93 (T1) and 0.94 (T2) in the present study.

#### Innovative Behavior

We used the five-item scale developed by Scott and Bruce (1994) to assess innovative behavior (e.g., “I search out new technologies, processes, techniques, and/or product ideas”). Five-point agreement Likert scales were used (1 = Completely disagree, 5 = Completely disagree). The internal consistency reliability of the scale was 0.85 (T1) and 0.90 (T2) in the present study.

#### Control Variables

People with different levels of personal resources may react differently to the same stressful situation. For example, Li et al. (2019) demonstrated that the older and married employees exhibited more presenteeism behavior. Other past research also found that female and managers reported sickness presenteeism more often than male and non-managers (Lu et al., 2013a; Sendén et al., 2016). We thus included gender (0 = female; 1 = male), age, marital status (0 = not married, 1 = married), and job position (0 = not manager, 1 = manager) in all analyses to exclude potential confounding factors.

### Strategy of Analysis

We used the SPSS 24 and PROCESS macro version 2.16.3 (Model 3) to test the moderated moderation effect. According to Hayes et al. (2017), PROCESS macro and hierarchical regression analysis produce consistent results, but PROCESS is able to directly estimate the mediated moderation effect. Bootstrapping with 5,000 samples was used to calculate bias-corrected confidence intervals. To take advantage of our two-wave data, we used independent variable (presenteeism) as measured at T1, and moderators (supervisory support, collegial support) and dependent variables as measured at T2 in all the following analyses. We further *controlled for the base-line levels of the dependent variables*, that is, exhaustion and innovative behavior as measured at T1. Before testing hypotheses, we conducted a confirmatory factor analysis (CFA) to verify the factor structure by confirming that each measure is loaded on a particular factor (Byrne, 2001). We also checked for the common method variance (CMV) bias, as our data are all self-reported (Podsakoff et al., 2003).

## Result

### Descriptive Analysis

Prior to the hypotheses testing, bi-variable correlations were computed, and results are shown in Table 1. Presenteeism (T1) positively correlated with exhaustion (T2). Both supervisory support (T2) and collegial support (T2) positively correlated with innovative behavior (T2). Exhaustion (T2) negatively correlated with innovative behavior (T2). None of the demographical characteristics correlated with presenteeism, though age positively correlated with exhaustion, supervisor support, and collegial support; and gender (male) and position (managers) correlated with innovative behavior.

**Table 1:** Correlations among study variables (*N* = 323).

	**Mean**	**SD**	**Gender**	**Age**	**Marriage**	**Position**	**T1SicPre**	**T2SicPre**	**T1SS**	**T2SS**	**T1WF**	**T2WF**	**T1Exhaustion**	**T2Exhaustion**	**T1InnoBeh**	**T2InnoBeh**
Gender	0.34	0.47	–													
Age	36.92	8.91	0.01	–												
Marriage	0.54	0.50	0.10	0.45^***^	–											
Position	0.29	0.45	0.17^**^	0.32^***^	0.24^***^	–										
T1SicPre	5.19	1.76	−0.08	−0.02	−0.00	0.01	–									
T2SicPre	5.55	1.75	−0.02	0.01	0.07	0.05	0.49^***^	–								
T1SS	16.61	4.89	−0.01	−0.18^***^	−0.16^**^	−0.05	0.04	0.08	–							
T2SS	15.62	4.89	0.06	−0.09	−0.00	0.03	−0.03	0.05	0.55^***^	–						
T1WF	21.33	3.94	−0.09	−0.12^*^	−0.06	−0.03	0.06	0.01	0.38^***^	0.18^***^	–					
T2WF	21.50	3.90	−0.09	−0.15^**^	−0.03	−0.00	0.02	−0.10	0.26^***^	0.23^***^	0.63^***^	–				
T1Exhaustion	21.17	10.89	−0.01	−0.16^**^	−0.10	−0.03	0.27^***^	0.16^**^	−0.13^*^	−0.08	−0.15^**^	−0.08^**^	–			
T2Exhaustion	21.08	11.20	0.01	−0.19^***^	−0.04	0.04	0.20^***^	0.26^***^	−0.05	−0.06	−0.12^*^	−0.12^*^	0.53^***^	–		
T1InnoBeh	18.42	2.90	0.24^***^	0.01	−0.03	0.17^**^	−0.08	−0.04	0.23^***^	0.11^*^	0.15^**^	0.00	−0.15^**^	−0.21^***^	–	
T2InnoBeh	18.42	3.00	0.22^***^	0.05	−0.04	0.14^**^	0.00	0.01	0.16^**^	0.20^***^	0.12^*^	0.17^**^	−0.11^*^	−0.18^***^	0.59^***^	1

*Marriage, marital status; Position, job position; SicPre, sick presenteeism; SS, Supervisory Support; WF, Workplace friendship*.

*Gender: 0 = female, 1 = male. Marital status: 0 = not married; 1 = married. Job position: 0 = employees; 1 = managers*.

* *p < 0.05*,

** *p < 0.01*,

*** *p < 0.001*.

### Hypothesis Testing

In order to test for discriminant validity, we conducted a CFA using AMOS 24. Combining data from both waves, we compared a hypothesized five-factor model (presenteeism, supervisory support, collegial support, exhaustion, and innovative behavior) with alternative models (in which these five factors were combined in different ways). The results displayed that the five-factor measurement model displayed a suitable fit to the data [χ^2^/*df* = 2.92, comparative fit index (CFI) = 0.91, root mean square error of approximation (RMSEA) = 0.08, standardized root mean square residual (SRMR) = 0.05] and outperformed any simpler representations of the data (*p* < 0.01 for all model comparisons). Self-report may increase the threat of CMV bias, a CMV test was performed following the procedure used by Williams et al. (1989); and this analysis revealed that the method factor did improve model fit (χ^2^/*df* = 2.742; CFI = 0.26; RMSEA = 0.07; RMR = 0.07), which is expected. Consequently, we calculated the variance explained by the method factor (Williams et al., 1989), accounting for only 13.5% of the total variance. This amount is less than the 25% threshold recommended by Williams et al. (1989). Thus, it was concluded that CMV was not a major concern in this study. Results of these tests are summarized in Table 2.

**Table 2:** Summary of the goodness-of-fit indices of the competing models (*N* = 323).

**Model**	**Model description**	**χ^**2**^ (df)**	**χ^**2**^/df (NC)**	**RMR**	**SRMR**	**GFI**	**NFI**	**CFI**	**RMSEA**
Model 1	5 factors	843.12 (289)	2.92	0.08	0.05	0.83	0.87	0.91	0.08
Model 2	3 factors	3,016.23 (296)	10.19	0.18	0.15	0.53	0.53	0.55	0.17
Model 3	1 factor	4,131.56 (299)	13.82	0.23	0.20	0.44	0.35	0.37	0.20
Model 4 (CMV check)	One latent method variable	721.41 (263)	2.74	0.07	0.20	0.84	0.89	0.92	0.07

*Five-factor model (Full model): presenteeism, supervisory support, collegial support, innovative behavior, exhaustion. Three-factor model: presenteeism, supervisory support+collegial support, innovative behavior+exhaustion. One-factor model: presenteeism+supervisory support+collegial support+innovative behavior+exhaustion*.

*SRMR, standardized root mean square residual; GFI, goodness of fit index; NFI, normed fit index; CFI, comparative fit index; RMSEA, root mean square error of approximation; CMV, common method variance*.

### Moderated Moderation Effects of Social Support at Work

We adopted Model 3 in PROCESS 2.16.3 to examine the moderated moderation effects with 5,000 bootstrap samples. This model was developed by Hayes et al. (2017) in order to estimate simultaneously the conditional effects and their significance based on 95% bootstrap confidence intervals. In the first step, the effects of individual characteristics and baseline dependent variable (i.e., exhaustion and innovative behavior at T1) were controlled. In the second step, we examined simultaneously the two-way and three-way interactions of presenteeism, supervisory support, and collegial support on exhaustion (T2) or innovative behavior (T2) separately. As shown in Tables 3, 4, the full model explained 32 and 42% of the variance in exhaustion [*F*_(12, 299)_ = 11.83, *p* < 0.001] and innovative behavior [*F*_(12, 299)_ = 18.04, *p* < 0.001], respectively.

**Table 3:** Moderated moderation effect of social support at work on the relationship between presenteeism and exhaustion (*N* = 323).

	**Exhaustion (T2 strain)**				
	**Coefficient**	**SE**	**Coefficient**	**95% CI**	
	**(B)**		**(β)**	**(LL, UL)**	
**Step 1: CV**					
Gender	−0.32^**^	1.18	−0.01	−2.64	2.00
Age	−0.22^***^	0.07	−0.18	−0.37	−0.08
Marriage	1.70	1.22	0.08	−0.70	4.10
Position	2.24	1.28	0.09	−0.29	4.76
T1Strain	0.49	0.05	0.48	0.39	0.60
**Step 2: X/W/Z**					
T1Presentism (X)	−4.97	4.67	0.07	−14.14	4.20
T2Supervisory support (W)	−0.87	1.87	0.01	−4.55	2.81
T2Collegial support (Z)	−1.34	1.17	−0.10	−3.64	0.95
X * W	0.25	0.35	−0.01	−0.44	0.95
X * Z	0.26	0.22	0.04	−0.17	0.70
W * Z	0.05	0.09	−0.03	−0.12	0.21
X * W * Z	−0.01	0.02	−0.04	−0.04	0.02
Total *R*^2^			0.32		
*F*			11.75^***^		

*Marriage, marital status; Position, job position*.

*Gender: 0 = female, 1 = male. Marital status: 0 = not married; 1 = married. Job position: 0 = employees; 1 = managers*.

** *p < 0.01*,

*** *p < 0.001*.

**Table 4:** Moderated moderation effect of social support at work on the relationship between presenteeism and innovative behavior (*N* = 323).

	**Innovative behavior (T2)**				
	**Coefficient**	**SE**	**Coefficient**	**95% CI**	
	**(B)**		**(β)**	**(LL, UL)**	
**Step 1: CV**					
Gender	0.60^*^	0.30	0.09	0.01	1.19
Age	0.04^*^	0.02	0.11	0.00	0.07
Marriage	−0.54	0.30	−0.09	−1.13	0.06
Position	−0.01	0.32	−0.00	−0.65	0.62
T1InnoBeh	0.55^***^	0.05	0.53	0.46	0.65
**Step 2: X/W/Z**					
T1Presentism (X)	−2.69^*^	1.17	0.11	−4.99	−0.39
T2Supervisory support (W)	−0.96^*^	0.48	0.13	−1.90	−0.02
T2Workplace friendship (Z)	−0.68^*^	0.29	0.18	−1.26	−0.11
X ^*^ W	0.17	0.09	−0.01	−0.01	0.35
X ^*^ Z	0.14^*^	0.06	0.02	0.03	0.25
W ^*^ Z	0.05^*^	0.02	0.04	0.01	0.09
X ^*^ W ^*^ Z	−0.01^*^	0.00	−0.09	−0.02	−0.00
Total R^2^			0.42		
*F*			18.02^***^		

*Marriage, marital status; Position, job position*.

*Gender: 0 = female, 1 = male. Marital status: 0 = not married; 1 = married. Job position: 0 = employees; 1 = managers*.

* *p < 0.05*,

*** *p < 0.001*.

The proposed relationship between presenteeism and exhaustion at T2 was not significant (Table 3); thus, our hypothesis 1a was not supported. Neither was the hypothesized three-way interaction of presenteeism × supervisory support × collegial support significant on exhaustion; thus, our hypothesis 2a was not supported. However, the pattern was different for innovative performance. There was a significantly negative effect of presenteeism on innovative behavior at T2 (Table 4), thus supporting our hypothesis 1b. The hypothesized three-way interaction of presenteeism × supervisory support × collegial support was also significant on innovative behavior (coefficient = −0.01, *p* < 0.05, 95% CI: −0.0162 to −0.0001), thus supporting our hypothesis 2b.

**Figure 2:**
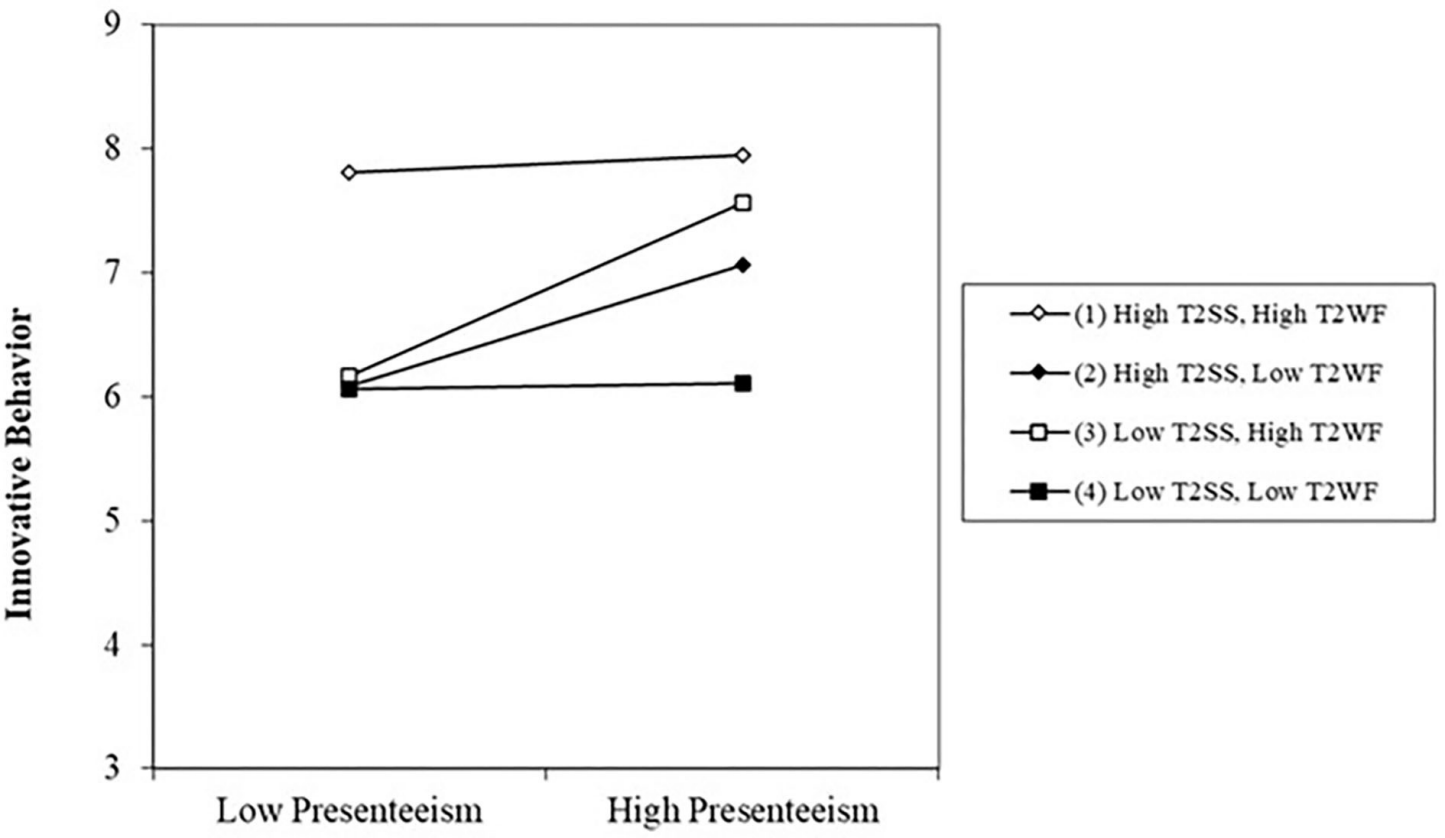
The 3-way interaction effect of presenteesim and social support at work on innovative behavior.

To reveal the moderation pattern, we applied the worksheet available online at http://www.jeremydawson.co.uk/slopes.htm (see also Dawson, 2014) to plot the simple effects for four subsamples as shown in Figure 2. Although slope tests revealed no pairwise significant differences among the simple regression lines, the overall pattern corroborated our hypothesis. Namely, when working through sickness, the innovative performance of those with high supervisory support coupled with high colleague friendship (subsample 1: abundance resources) were *the least* affected. Those having minimal resources (subsample 4: low on both supervisory and collegial support) suffered the most severe blow on innovative performance under sickness conditions, showing as the lowest line in the group. Contrasting the pair of lines for those with at least one source of support available (subsample 2: high supervisory support; subsample 3: high collegial support) against the bottom line (subsample 4), we noted that the negative effect of presenteeism on innovative performance was somewhat reversed (i.e., Lines 2 and 3 went upward). Thus, our theorized compensatory effect of resources was tentatively confirmed under the sickness presenteeism condition, though the substantial benefit of supervisory support and collegial support seemed equivalent. Overall, the pattern of the three-way interaction supported our hypothesis 2b; that is, in sickness presenteeism, the employee innovative behavior was at the highest level when both supervisory support and collegial support were high.

## Discussion

### Theoretical Contribution

The objective of this study is to clarify the lasting effect of presenteeism on employees' well-being and innovative behavior, incorporating the joint effect of dual source of social support at work on alleviating the potential damages of the presenteeism behavior. Contributing to the inclusiveness of scientific contents in the post-pandemic era, our study was conducted in the under-studied Asian populations who nonetheless are more prone to commit sickness presenteeism and suffer worse consequences of the behavior (Lu et al., 2013a). In the time frame of 6 months, we did find lasting damaging effects of presenteeism on employees' future innovative performance, though not on exhaustion. Consistent with the Hansen and Andersen (2008) findings, we confirmed that working under illness is indeed harmful for innovative performance, and such damage was not transient, as it lasted for at least 6 months. However, it is worth noting that we did not find a lasting damaging effect of presenteeism on employees' future exhaustion, contrary to some existing studies (Demerouti et al., 2009; Lu et al., 2013b), but consistent with other studies (e.g., Lu et al., 2014). The mixed findings may be due to different measures of presenteeism (Demerouti et al., 2009), or different time frames used (Demerouti et al., 2009; Lu et al., 2013, Lu et al., 2014), or intervening psychological mechanisms, such as motivational regulation (Dew et al., 2005) and organizational support (Garrow, 2016). Clear research on the consequences of presenteeism for attitudinal, affective, and motivational processes is sparse (Miraglia and Johns, 2016), and we need more studies to understand the development of such consequences over time.

More importantly, we found three-way interactive effects of presenteeism, supervisory support, and collegial support on employees' innovative performance. As the analysis of the moderated moderation model showed, when working under illness, employees displayed the best innovative performance with high levels of both supervisory and collegial support. We also found that employees benefited from having at least one source of support, from either the supervisors or the colleagues. Our findings are consistent with previous studies; for example, Brunner et al. (2019) found that job and personal resources can buffer the negative effects of job stressors on health-related productivity losses. Furthermore, they also found that the compensatory effect of job resources for employees with low personal resources in the high stress situations. Therefore, we confirmed the rarely investigated compensatory effects of resources in the stressor–strain relationships, by disentangling the joint effects of different resources.

Above all, this study contributes to the flourishing presenteeism literature and COR theory in two ways: First, adding to the scarce research on the relationship between presenteeism and employee innovation (Hansen and Andersen, 2008; Fan and Lu, 2020), we confirmed that the lasting negative effect of working under illness is indeed harmful for employee innovative performance. As employee innovative behavior is critical for firm innovation and competitiveness (Amabile et al., 1996; Yuan and Woodman, 2010), such a key aspect of the employees' job performance should be included in evaluating the consequences of presenteeism. Our finding thus contributes to substantiating the “bad presenteeism” scenario (Cooper, 1996; Hemp, 2004; Demerouti et al., 2009; Lu et al., 2013b), extending its negative outcomes to the future innovative performance.

Second, we extend the resource caravans perspective of the COR theory and confirm the joint effects of supervisory and collegial support in the relationship between presenteeism and innovative behavior. This is in line with the COR proposition that resources gain are even more important when facing resource loss (Hobfoll, 2012). Our pattern of the three-way interaction (Figure 2) also corroborates Hobfoll and Leiberman (1987) finding that having more than one type of resource, whether personal resources or social resources, may be better than having one only. This pattern of resource value as a function of *source* may be more pronounced in the Asian societies of “the Confucian Circle,” including the mainland China, Taiwan, Hong Kong, Japan, Korea, and Singapore. This is because the Confucian tradition puts great emphasis on *Guanxi* (social relationships) as the fiber of the society. More importantly, theoretical analysis on *Guanxi* regarding the characteristics of the collectivist culture postulates that different relationships are used to satisfy different needs and thus have different values for adaptation (Hwang, 1997). The two broad genres of relationships in Confucian societies, vertical and horizontal, manifest in the supervisor–subordinate and co-workers interactions at work. The vertical relationships, namely, those with the authorities in the society, seniors in the family, and superiors at work, help secure valuable resources, prospects and advancement; the horizontal relationships, namely, those with people of the same social gradient in the society, the same generation in the family, and peers at work, help satisfy psycho-emotional needs of belongingness and intimacy (Triandis and Gelfand, 1998). As a pillar of the Confucian ethics, the Five Cardinal Relationships (*Wu Lun*) dictate that the vertical relationships command much greater eminence that the horizontal relationships for the society and the individual (Hwang, 1997); thus, the Chinese societies are dubbed vertical collectivism (Triandis and Gelfand, 1998).

In the collectivist culture where the present study was conducted, vertical relationship (e.g., supervisory support) and horizontal relationship (e.g., collegial support) are fundamentally different but equally valuable social resources to people (Hwang, 1997). In the Chinese workplace, supervisory support is a general indicator of good leadership–member exchange (LMX), which emphasizes leaders' provision of resources and support at the individual level, rather than amending organizational matters such as the imposition of detriments on employees taking sick leave (Wang et al., 2018). Employees with high-quality relationships with their leaders (LMX) have more job resources to deal with work stress and demands (Cheng et al., 2012). Line management support is of utter importance in making presenteeism a “sustainable choice” for employees should they be willing to do so (CIPD, 2016). On the other hand, collegial support is also a vital work resource to mitigate the noxious effects of presenteeism on employees' well-being and productivity (Dew et al., 2005). Our findings thus confirm the advantageous effect of having the abundance of resources at one's disposal in a challenging work situation (i.e., having two is the best scenario); it also seems that the resource compensatory effect as proposed by the COR occurred for both collegial support and supervisory support (i.e., having one is better than none). This pattern of nuanced disparity in the utility of support from different sources, and the underlying dynamism of mobilizing different types of work support deserves further exploration, for example, the joint effects of personal resources and job resources on the stressor–strain relationships (Hobfoll and Leiberman, 1987; Brunner et al., 2019).

### Managerial Implications

In the West, an understanding supervisor may be able to relieve subordinates from fear of leaving a bad impression when taking sick leaves; thus, there is no need to use presenteeism as either a career-protecting or a career-promoting tactic. Baker-McClearn et al. (2010) discovered in a qualitative study in the UK that supervisory support was pivotal for employees deciding not to come to work when ill. However, factors involved in deciding to turn up to work while ill may be very different for the Chinese employees. As the Chinese culture puts so much emphasis on hardworking and perseverance, even with a sympathetic direct line supervisor, employees may still push themselves to work to present a good image to a wider audience, including co-workers, managers of higher levels, and even customers. Thus, when supervisory support is absent or non-effective, supplementing it with other resources such as collegial support may change the game, especially when the going gets tough in the post-pandemic times. Strengthening team cohesion was found to enhance member satisfaction and performance in the demanding Chinese work environment (Lu and Fan, 2017). Thus, to constructively manage presenteeism and protect employees' well-being and performance, line managers and co-workers need to be educated and trained to play key roles in sustaining integration at work. For instance, managers still need to be aware that work overload precipitates presenteeism, harming employees' job outcomes. Furthermore, organizations and supervisors should nurture good leader–subordinate relationship as well as coworker relationships, to foster emotional support and work-directed interventions, such as setting work replacements to ensure supplement when an employee is ill.

In addition, although we found evidence that social support played an important role in attenuating some long-lasting noxious effects of presenteeism, organizations and managers still need to be aware that sickness presenteeism is harmful to employees' job performance and well-being, both immediately and in the long run. Thus, to tackle the problem at its root, organizations should invest in health promotion programs and work-directed interventions, such as setting work replacements. Amid economic recession and prevailing hardships at the wake of the triple pandemic, caring for employees' well-being and quality of work creates a more poignant impression of good employer responsibility and corporate commitment.

### Limitation and Future Directions

The current study is subject to some limitations and opens up new avenues for further research. First, we adopted self-report measures, which may increase the threat of CMV bias (Podsakoff et al., 2003). In an effort to minimize such bias, we adopted a panel design and measured all study variables twice, to separate the independent variables (presenteeism), moderators (supervisory support and collegial support), and dependent variables (exhaustion and innovative behavior) in time. To get more comprehensive knowledge, we suggest future studies should consider including objective measurements of job performance. Second, we used Hayes' PROCESS (Hayes, 2013) to test the simultaneously intervention effects of supervisory support and collegial support on the relationship between presenteeism and outcomes. Our proposed moderated moderation model was supported for innovative performance, but not for exhaustion. Future research may measure the concept of burnout including other dimensions (i.e., depersonalization or personal accomplishment) (Taris et al., 2005) and further explore the interactive effects of collegial support and supervisory support on a wider range of strains and outcomes.

Third, another limitation is the fact that we did not assess any personal resources. Previous studies on innovation and creativity have found the protective role of numerous personal variables, such as mindfulness (Montani et al., 2019). However, the broad concept of resource caravans proposes that employee would utilize multiple resources at a time, depending on the demands and the context (Hobfoll et al., 2018). For instance, Stetz et al. (2006) found that self-efficacy and social support had joint effects on the stressor–strain relationships. Future research could investigate the simultaneous interaction effects of a wide range of individual or organizational resources in the context of working under illness. Fourth, we did not include any COVID-19-related variables in the study. As Taiwan has been very fortunate in escaping from the devastating impact of the pandemic and largely successful in holding on to a normal life with the cost of strictly sealing its borders and thoroughly reinforcing quarantines, it was deemed unnecessary to include any COVID-19-related variables, given the small variations. In hindsight, it is wiser to directly assess the COVID-19-related individual exposure as control variables to rule out any potential individual differences. Finally, although our results confirmed the resources compensatory effects of the dual social support in the presenteeism–outcomes relationship for Chinese workers, we cannot rule out the possibility of artifacts, as our study was situated in the Confucian culture, where work is given a high priority. Social capital is a vital resource for Taiwan employees in stress coping, regardless of the source (supervisory support or collegial support), and thus may equally help sustain the employees' innovative performance when working ill. More research is needed to replicate and understand the moderated moderation effects of supervisory and collegial supports in other cultures.

To conclude, sickness presenteeism did hamper employees' innovative behavior even measured in 6 months after the behavior. Mobilizing social resources at work, namely, supervisory support and collegial support, could mitigate the lasting damages of working with illness. Employees with the abundance of resources fared the best; however, social resources compensate for one another in coping, leaving the ones with the minimal resources to suffer the worst. As the going gets tough and dark night seems long, creating, nurturing, deploying, and utilizing resources may hold the key for thriving, not just surviving.

## Data Availability Statement

The raw data supporting the conclusions of this article will be made available by the authors, without undue reservation.

## Ethics Statement

The studies involving human participants were reviewed and approved by Research Ethics Committee, National Taiwan University. Written informed consent for participation was not required for this study in accordance with the national legislation and the institutional requirements.

## Author Contributions

LL: conceptualization and funding acquisition. LL and JC: methodology and writing—original draft preparation. JC: formal analysis. CC: resources. LL, JC, and CC: writing—review and editing. All authors have read and agreed to the published version of the manuscript.

## Conflict of Interest

The authors declare that the research was conducted in the absence of any commercial or financial relationships that could be construed as a potential conflict of interest.
